# The Effect of Tourniquet Duration on Pain, Bleeding, and Functional Outcomes in Total Knee Arthroplasty

**DOI:** 10.7759/cureus.34606

**Published:** 2023-02-03

**Authors:** Hikmet Çi̇nka, Alparslan Yurtbay, Furkan Erdoğan, İsmail Büyükceran, Hüseyin S Coşkun, Yılmaz Tomak

**Affiliations:** 1 Orthopedics and Traumatology, Karasu State Hospital, Sakarya, TUR; 2 Orthopedics and Traumatology, Samsun Education and Research Hospital, Samsun, TUR; 3 Orthopedics and Traumatology, Sabuncuoğlu Şerefeddin Research and Training Hospital, Amasya, TUR; 4 Orthopedics and Traumatology, Samsun Ondokuz Mayis University, Samsun, TUR

**Keywords:** outcomes, duration, tourniquet, total knee arthroplasty, gonarthrosis

## Abstract

Objective

The aim of this study was to evaluate the effects of tourniquet use on perioperative blood loss, pain, and functional and clinical outcomes.

Patients and methods

This is a prospective study that included 80 knees who underwent total knee arthroplasty. The patients were separated into two groups: those with a tourniquet used throughout the entire surgical procedure and those where the tourniquet was only used during the cementation procedure. In the postoperative period, the pain levels of the patients were evaluated using a visual analog scale (VAS), and the functional results were evaluated with knee range of motion measurement, the Western Ontario and Mcmaster Universities Osteoarthritis (WOMAC) index, the Knee Injury and Osteoarthritis Outcome Score (KOOS), the Kujala Patellofemoral Scoring System, and the Oxford Knee Scoring system. The patients were examined in the early postoperative period and again in the 12th week, including possible complications that may develop postoperatively.

Results

In the early postoperative period, a greater hemoglobin decrease and calculated blood loss values, better functional clinical results, and better knee range of motion were determined in the group with a tourniquet applied only during the cementation, and the swelling in the knee was less (p<0.05). However, the difference between the two groups had disappeared by the postoperative 12th week. There was no significant difference in respect of complications.

Conclusion

Limiting the duration of tourniquet use during total knee arthroplasty has the significant advantage of providing better functional results with less pain in the early postoperative period.

## Introduction

Primary osteoarthritis is a degenerative disease characterized by loss of joint cartilage, inflammation of synovial tissue, and the formation of osteophytes. There are both conservative and surgical treatment options for knee osteoarthritis (gonarthrosis). When conservative methods are insufficient, total knee arthroplasty (TKA) is a frequently selected and performed procedure.

Tourniquets are commonly used by surgeons in TKA. The use of a tourniquet provides better visualization of the surgical field, shortens the operating time, and reduces perioperative blood loss [1]. However, there are several questions related to the reliability and efficacy of tourniquet use. There are studies in the literature that have reported that there could be disadvantages, such as increased postoperative pain in the thigh [2], nerve paralysis, rhabdomyolysis, swelling in the extremity, vascular injuries, and subcutaneous fat necrosis [3]. Therefore, whether the use of a tourniquet is useful or not in TKA remains a matter of debate. 

The patients in this study were separated into two groups: those where a tourniquet was used throughout the operation and those where a tourniquet was applied only during the cementation procedure. The aim of this prospective study was to examine the effect of the duration of tourniquet use in total knee arthroplasty operations on hemoglobin values and postoperative pain, clinical functional results, and possible complications.

## Materials and methods

The study included 80 knees out of 72 patients who presented with complaints of knee pain and were diagnosed with gonarthrosis. Inclusion criteria was patients with primary gonarthrosis, and adults aged 18 years or over. The exclusion criteria included the inability or refusal to provide informed, written consent; revision TKA; peripheral vascular disease; a history of bleeding diathesis; and a history of musculoskeletal infection of the limb.

In 38 of the 80 knees, a tourniquet was applied at 300 mmHg pressure before starting the operation, and the whole surgical procedure was performed under a tourniquet. In 42 knees, the tourniquet was used only at the stage of cementation.

Tranexamic acid (TXA) at a dose of 10mg/kg was administered intravenously to both groups before starting the surgery. In both groups, surgery was performed with a standard surgical technique using a medial parapatellar approach. The same TKA instrumentation was used in all patients. In the group defined as the partial tourniquet group, a tourniquet was used only at the stage of cementation, which lasted for an average of 15-20 minutes. An aspirative drain was used in all the patients and was removed at a mean of 36-48 hours postoperatively. As medical prophylaxis against deep vein thrombosis (DVT), low-molecular weight heparin was used.

Follow-up of the patients was recorded for 12 weeks. The values recorded in the examinations preoperatively, in the early postoperative period, and at 12 weeks were compared.

The patients were evaluated in respect of age, gender, body mass index (BMI), comorbidities, gonarthrosis etiology, surgery-related complications developing postoperatively, the change in preoperative and postoperative hemoglobin values, the change in knee circumference measurements, the change in knee joint range of movement (ROM), visual analog scale (VAS) scores, functional evaluation tests (Knee Injury and Osteoarthritis Outcome Score (KOOS), the Oxford Knee Score, the Kujala Patellofemoral Scoring System), the type of anesthesia used, and the number of blood products used postoperatively. The patients were evaluated preoperatively, on postoperative day three and 12 weeks postoperative.

The hemoglobin concentration levels of the patients were measured preoperatively and on the 3rd day postoperatively. Using the hemoglobin balance method, the amount of blood loss was calculated and compared between the groups. The amount of blood collected in the drain and from the dressings was calculated as the postoperative blood loss.

This study was designed as a single-center prospective study. All patients were randomly divided into two groups using the research randomizer. Verbal and written informed consent was obtained from all the patients included in the study.

The study was approved by the local Clinical Research Ethics Committee (B.30.2.ODM.0.20.08/09-73-102). In this prospective study, all procedures and practices are in accordance with the ethical standards of the national/ institutional research committee and the 1964 Helsinki declaration.

Data obtained in the study were analyzed statistically using SPSS version 20.0 software (IBM Inc., Armonk, New York). The conformity of data to normal distribution within and between groups was assessed with visual (histogram and probability graphs) and analytical methods (Kolmogorov-Smirnov/Shapiro-Wilk tests). In the comparisons of two independent groups conforming to a normal distribution, the independent paired samples t-test was applied, and for two groups not showing normal distribution, the Mann-Whitney U-test was used. A value of p<0.05 was accepted as statistically significant.

## Results

The patients comprised 69 females and three males with a mean age of 66.9 years (range 51-85 years). The demographic data and baseline values of the groups are shown as mean ± standard deviation (SD) values in Table 1. In the comparison of the baseline values of the groups, no statistically significant difference was determined with respect to gender, BMI, operated side, knee joint ROM, knee circumference measurements, KOOS, Kujala knee scores, and hemoglobin values. In the patients operated on under a tourniquet, the preoperative knee circumference measurements were greater, and the VAS scores were lower compared to the other group.

**Table 1:** Comparisons of the baseline demographic data and scores of the groups VAS - visual analog scale, WOMAC - Western Ontario and McMaster Universities Arthritis Index

	Without tourniquet, n:42	With tourniquet, n:38	p-value (between the groups)
Gender, male:female	2:40	1:37	0,370
BMI	32.96±5.40	34.03±5.57	0.345
Operated side, right:left	23:19	20:18	0.850
Knee circumference measurement	44.40±5.56	48.44±8.18	0.015*
Joint range of movement	91.42±32.23	101.31±23.06	0.181
VAS	8.35±1.49	7.57±1.58	0.022*
WOMAC	66.92±18.27	70.89±12.70	0.525
Kujala	29.28±11.75	33.94±14.66	0.119
Hemoglobin	12.08±1.56	12.53±1.25	0.129

An increase in knee circumference was observed in both groups in the early postoperative period, and this change was statistically significantly greater in the tourniquet group (p<0.05). In the comparison of the change in the knee circumference values at 12 weeks postoperatively compared to the baseline values, no statistically significant difference was determined between the groups (p=0.590).

On postoperative day three, there was determined to be a statistically significantly greater decrease in knee ROM in the tourniquet group (p<0.05), and at 12 weeks, the change compared to the baseline values was not found to be statistically significant (p=0.191).

On postoperative day three, there was determined to be a statistically significantly greater increase in VAS score in the tourniquet group (p<0.05), and at 12 weeks, the change compared to the baseline values was not found to be statistically significant (p=0.149) (Table 2).

**Table 2:** Comparisons of the changes in the parameters of the groups from preoperative to postopertive day three and postoperative 12 weeks VAS - visual analog scale, KOOS - Knee Injury and Osteoarthritis Outcome Score, WOMAC - Western Ontario and McMaster Universities Arthritis Index

	Preoperative			Change on postoperative day 3			Change at 12 weeks postoperative		
	Without tourniquet	With tourniquet	p-value	Without tourniquet	With tourniquet	p-value	Without tourniquet	With tourniquet	p-value
VAS	8.35±1.49	7.57±1.58	0.022*	-1.73±2.16	-0.34±1.97	0.004*	-4.52±1.59	-4.00±1.91	0.149
Knee circumference measurement	44.40±5.56	48.44±8.18	0.015*	3.26±1.12	4.23±1.90	0.008*	0.90±1.39	0.39±4.16	0.59
Joint range of movement (degrees)	91.42±32.23	101.31±23.06	0.181	-11.90±41.23	-48.07±39.97	0.001*	11.78±33.08	-6.83±28.39	0.191
KOOS Score	23.61±15.07	27.52±10.27	0.033*	19.28±14.46	11.15±11.86	0.012*	43.11±13.27	38.26±14.52	0.122
WOMAC Score	66.92±18.27	70.89±12.70	0.525	-17.54±13.23	-7.86±8.86	0.001*	-42.73±15.73	-41.94±14.56	0.747
Kujala Patellofemoral Score	29.28±11.75	33.94±14.66	0.119	12.28±9.14	5.52±7.75	0.002*	35.73±11.57	30.34±15.34	0.078
Oxford Knee Score	12.45±6.05	12.97±5.17	0.553	9.83±7.79	5.76±5.13	0.031*	20.61±7.02	18.18±7.45	0.334

In the evaluation of the functional scores on postoperative day three (KOOS, WOMAC, Kujala, Oxford Knee Score), the changes in the functional scores were determined to be statistically significantly better in the group applied with tourniquet only during cementation (p<0.05). When the groups were evaluated at 12 weeks, no statistically significant difference was found in respect of the change compared to the baseline values (p>0.05) (Table 2).

When the decrease in hemoglobin was calculated from preoperative to postoperative, there was a statistically significant difference (p<0.05) (Table 3). The decrease in hemoglobin was determined to be statistically significantly greater in the group applied with a tourniquet only during cementation (p<0.05) (Table 3). The mean amount of transfusion was calculated as 0.20 units. No significant difference was determined between the two groups in respect of the requirement for blood transfusion (p>0.05).

**Table 3:** Hemoglobin values, calculated blood loss, and amount of blood collected in the drain

	Without tourniquet, n:42	With tourniquet, n:38	p-value (between groups)
Preoperative hemoglobin value (g/dl)	12.08±1.56	12.53±1.25	0.129
Postoperative hemoglobin value (g/dl)	10.16±1.57	11.21±1.16	0.006*
Change in the hemoglobin value (g/dl) in the early postoperative period	-1.92±0.89	-1.32±0.68	0.019*
Calculated blood loss (V_total_=ml)	679.92±283.48	478.35±239.78	0.002*
Amount of blood collected in the drain	301.25±46.92	332.50±51.99	0.053

The amount of blood collected from the drains was evaluated to compare postoperative blood loss, and no significant difference was determined between the groups (p>0.05) (Table 3).

Deep vein thrombosis that was symptomatic or required treatment did not develop in any patient. In the tourniquet group, revision surgery was performed in one patient because of infection, and minor wound problems developed in two patients, which were successfully treated with debridement and wound dressings. In the group applied with tourniquet only during cementation, minor wound problems developed in two patients, with discharge determined in one, which was treated with irrigation.

## Discussion

Tourniquets have been used in knee arthroplasty surgery with the aim of creating a relatively blood-free operating field, to provide better visualization of structures, and to reduce intraoperative blood loss [1]. However, despite widespread use, there are complications associated with tourniquet use. It is thought that these conditions can be explained by hypoxia and reperfusion [3, 4].

There is limited data about the effect of a limited tourniquet duration strategy on the clinical outcomes of primary TKA. The hypothesis of this study was that tourniquet use during only the cementation procedure of TKA could increase intraoperative blood loss but improve knee function and could also decrease the possibility of thromboembolism and other minor wound complications.

Swelling of the lower extremity occurs in the majority of TKA patients. Previous studies have shown that damage to the blood and lymph vessels, increased tissue permeability, extravasation of blood cells to tissue, and the expression of inflammatory factors are related to swelling in the extremity after TKA [5]. In a study by Li et al. [6], the free hemoglobin levels were determined to be significantly greater in the tourniquet group than in the group without tourniquet. This meant that hemocytolysis was greater in the tourniquet group. The mechanism of occult blood loss is generally accepted as loss caused by the accumulation of residual blood in the joint, extravasation to the tissues, and hemolysis. In literature, it has been shown that tourniquet use increased occult blood loss, and this was determined to be greater in the group with a pneumatic tourniquet on the third and 14th days postoperatively [6]. Li et al. [6] showed that in measurements made with the palm of the hand, there was a greater area of ecchymosis and more swelling developed in the extremity in the tourniquet group.

Changes in knee circumference measurements were evaluated in the current study, and while there was seen to be more swelling in the tourniquet group in the early postoperative period, at the 12th postoperative week, there was no significant difference. It was thought that the development of more swelling in the tourniquet group in the early postoperative period was due to local reactive hyperemia associated with tourniquet use resulting in greater residual blood accumulation in the knee joint and greater extravasation. While these changes created a difference in the early postoperative period, they were observed to have been eliminated with compensatory mechanisms in the long term.

There is significant evidence that tourniquet use increases postoperative pain and deteriorates function [3, 7]. Vandenbussche et al. [7] compared patients who underwent TKA operation with and without a tourniquet. The pain scores at six hours postoperatively were found to be lower in the group without a tourniquet, but at 24 and 48 hours, no significant difference was determined. Similarly, Pfitzner et al. [8] found an increase in pain in the early postoperative period in the tourniquet group. In the results of the current study, the VAS scores were determined to be lower in the patients where a tourniquet was only used during the cementation period, but this was only valid for the early postoperative period. When the change from baseline to 12 weeks postoperatively was examined, this advantage was no longer seen. In literature, the reason for this has been thought to be the post-ischaemic swelling of soft tissue and the direct effect of trauma of the tourniquet on nerve structures and soft tissues [3, 6, 7, 9]. This is supported by data showing that a tourniquet inflated at a lower pressure and used for a shorter time results in a decrease in postoperative pain [10].

In a study by Ritter of 4727 cases of TKA, the most important factor determining postoperative joint ROM was reported to be preoperative ROM [11]. Other factors affecting joint ROM are the surgical technique, the type of prosthesis used, age, etiology, gender, preoperative tibiofemoral alignment, and postoperative rehabilitation. Previous studies have shown that with decreased duration of tourniquet use, there was an improvement in early postoperative ROM [3, 7, 12]. In the current study, the tourniquet group had lower ROM in the early postoperative period, and there was no significant difference at 12 weeks. This was thought to be a result of the local pressure or local quadricipital rhabdomyolysis effect caused by the tourniquet having recovered by the 12th week. It was also thought that the post-ischaemic swelling of soft tissue, the greater occult blood loss within the joint, and the direct effect of trauma on the soft tissues allowed better joint ROM results to be obtained in the early postoperative period in the group with tournquet used only during cementation.

There are approximately 30 scoring systems that are used in the evaluation of knee damage [13]. Just as in many studies related to TKA in literature, the KOOS, WOMAC, Kujala Patellofemoral Score, and Oxford Knee score were used in the current study to evaluate the outcomes. In a study by Ejaz et al. [14], the clinical results and KOOS scores were determined to be better in the group without a tourniquet. 

Mittal et al. [15] found no significant difference between the two groups in respect of the Oxford Knee Score values. However, the scores were only examined preoperatively and in the 10th week postoperatively, with no follow-up in the early postoperative period. As there was no difference in the current study at 12 weeks, this was a common finding of both studies.

The knee functional scores (KOOS, WOMAC, Kujala Patellofemoral Score, and Oxford Knee Score) were found to be better in the early postoperative period in the group with tourniquet only during cementation, and at 12 weeks postoperatively, the improvement in functional results was determined to be similar in both groups. The better early results were thought to have been affected by having less postoperative pain, less swelling in the extremity, and less local effect of the tourniquet. The decrease in pain occurring with a shorter duration of tourniquet use has positive effects on postoperative rehabilitation and results in better functional scores.

One of the scores examined in the current study was the Kujala patellofemoral scoring system. Patellofemoral pain is a frequently seen knee problem that has a significant effect on the functional capacity and quality of life of patients. The Kujala patellofemoral score is a tool that provides a functional evaluation of knee complaints associated with the patellofemoral structure. No study could be found in the literature that has compared patellofemoral pain with tourniquet duration. It was thought that the difference in the score change between the two groups in the early postoperative period could be attributed to greater bleeding within the joint in the tourniquet group, as more intra-articular bleeding and increased swelling can increase patellofemoral pain.

Several studies have examined the relationship between tourniquet use and blood loss [1, 16, 17]. In the current study, the blood loss values were one of the primary evaluation results. Tourniquet use is thought to decrease blood loss. Several studies have evaluated this by using and not using a tourniquet during TKA, but the results are not clear. A widely used method to determine blood loss is the Gross formula, which is used to evaluate the change in the primary factor of hemoglobin [18]. When interpreting findings in the literature, it is important to bear in mind the various forms of blood loss and the definitions of these. In the current study, the amount of blood loss was calculated using the hemoglobin balance method [19]. In this study, the decrease in hemoglobin values and calculated blood loss were determined to be statistically significantly less in the tourniquet group (p<0.05). Alçelik et al. [16] calculated total blood loss and reported that tourniquet use reduced blood loss. In another study, Kato et al. [20] found a significant decrease in mean blood volume in the group without a tourniquet. However, no details of the calculation method were given in that study. In a meta-analysis by Smith et al. [1], intraoperative blood loss was determined to be greater in a group where a tourniquet was not used, but no difference was determined between the two groups in respect of postoperative blood loss measured from the drains or in transfusion rates. Vandenbussche et al. [7] reported that the calculated total blood loss was reduced with tourniquet use, but there was no difference between the two groups with respect to the amount of blood collected from the drains postoperatively. In line with the literature, despite the calculated blood loss being higher in the tourniquet group in the current study, there was no statistically significant difference between the two groups when the measured drain amounts were compared (p>0.05). 

There are studies showing an increased potential risk of DVT associated with thromboembolism causing potential damage to blood vessels and venous stasis in the lower extremity in a long-term tourniquet group [16, 20]. Zhang et al. [21] showed a higher incidence of thrombosis and thromboembolic events when a tourniquet was used in TKA [3, 12, 22]. Thrombus formation is related to the triad of venous stasis, endothelial damage, and hypercoagulability [23]. Tourniquet use may cause venous stasis, endothelial damage through direct trauma, and potential damage in calcified blood vessels. Zahavi et al. [24] reported that ischemia due to tourniquet use increased plasma beta-thromboglobulin and plasma thromboxane B2 levels and thus increased the risk of thrombosis in TKA patients. In addition, Katsumata et al. [25] found that tourniquet use during TKA could support the local expression of neutrophil elastase from neutrophils together with reactive oxygen species, and this could contribute to the development of DVT, pulmonary embolism (PE), and tissue damage. No symptomatic DVT was determined in any patient in the current study. Prophylaxis with low-molecular-weight heparin and early physiotherapy may have contributed to this result. No tests were applied to determine asymptomatic embolic events in the current study, which could explain the lack of difference between the groups. The duration of the study may have been another limitation, as there may have been a greater risk of seeing DVT if the follow-up period had been longer.

Various other complications associated with tourniquet use have been reported in the literature. In some studies, changes caused by tourniquet use have been reported in intraoperative patellofemoral observation [26, 27].

Superficial wound site problems were determined in two patients in each of the groups of the current study. Revision surgery was applied to one patient in the tourniquet group because of infection. In one of the patients in the group applied with a tourniquet only during cementation, irrigation was performed with an initial diagnosis of periprosthetic infection. No significant difference was determined between the groups in respect of superficial wound site problems or infection (p=0.321).

In the literature, there is still no consensus on some points regarding the tourniquet duration. One of the limitations of the study is that a larger patient group may be needed. Another limitation of this study is the lack of routine imaging to detect non-symptomatic cases of DVT.

## Conclusions

In light of these findings, it can be concluded that limiting the duration of tourniquet use during TKA has the significant advantage of providing better functional results with less pain in the early postoperative period. Thus patients can participate in rehabilitation exercises more effectively at an earlier period, which can make recovery easier for the patients. Therefore we recommend limiting the use of a tourniquet in TKA surgery.
